# Application of a novel osteotomy instrumentation as a substitute tool in total hip arthroplasty

**DOI:** 10.1186/s12891-022-05404-8

**Published:** 2022-05-11

**Authors:** Yuehao Hu, Jingwei Zhang, Ziyang Sun, Degang Yu, Huiwu Li, Zhenan Zhu, Yuanqing Mao, Mengning Yan, Zanjing Zhai

**Affiliations:** 1 Shanghai Key Laboratory of Orthopaedic Implants, Department of Orthopaedic Surgery, Shanghai Ninth People’s Hospital, Shanghai Jiao Tong University School of Medicine, Shanghai, 200011 People’s Republic of China; 2 Department of Orthopedics, Shanghai Jiao Tong University Affiliated Sixth People’s Hospital, Shanghai, 200233 People’s Republic of China

**Keywords:** Total hip arthroplasty, Osteotomy instrumentation, Femoral neck osteotomy

## Abstract

**Background:**

Mechanical failure, power shortage, and inadvertent contamination of the oscillating saw occasionally occurs in actualizing femoral neck osteotomy during total hip arthroplasty (THA); however, no appropriate alternative solution is currently available. This study aimed to introduce a novel osteotomy instrumentation (fretsaw, jig, cable passer hook) as a substitute tool while the oscillating saw was unavailable during THA.

**Methods:**

This study included 40 patients (40 hips) who underwent femoral neck osteotomy during primary THA using the new osteotomy instrumentation (*n* = 20) and the oscillating saw (*n* = 20). Clinical data and intraoperative findings of all patients were evaluated.

**Results:**

The mean osteotomy time was 22.3 ± 3.1 s (range, 17–30 s) and 29.4 ± 3.7 s (range, 25–39 s) in the oscillating saw group and in the new osteotomy instrumentation group, respectively (*P* < 0.001). The Harris Hip Score (HHS) improved in both groups; the mean HSS was 82.3 ± 2.5 and 83.3 ± 3.5 in the oscillating saw group and new osteotomy instrumentation group at 6 months after surgery, respectively (*P* = 0.297).

**Conclusions:**

The original osteotomy instrumentation can be an ideal substitute tool for femoral neck osteotomy in THA, especially when the oscillating saw is unavailable or malfunctioning.

## Background

Total hip arthroplasty (THA) is generally considered one of the most effective and safe procedures for relieving pain and restoring function in patients with hip joint disorders [1, 2]. More than 1 million hip arthroplasties are performed annually worldwide for severe osteoarthritis, osteonecrosis of the femoral head, and developmental dysplasia of the hip [3]. The number of cases of THAs in China is approximately 40,000 in recent years, which increases by 25%–30% each year [4]. Oscillating saws are widely used in orthopedics and have become a foundational tool for THA due to its high cutting efficiency and accuracy [5]. However, intraoperative complications occasionally occur due to its mechanical malfunction. Problems associated with oscillating saws include machine contamination and power shortages during femoral neck osteotomy in THA. Substituting the oscillating saw with a new one may prolong the operation time, which is associated with an increased risk of surgical infection [6]. In addition, most oscillating saws are heavy, and manual handling requires considerable training to avoid overshooting and consequently damaging the surrounding soft tissue and neurovascular bundles [7]. In several cases, orthopedists use a fretsaw or a chisel as a substitute tool for femoral neck osteotomy during THA. However, research studies on these osteotomy tools are limited.

In this study, we designed a compact, affordable, and efficient reusable femoral neck osteotomy instrumentation based on a fretsaw, jig, and cable passer hook. This study aimed to introduce and evaluate this novel osteotomy instrumentation as a substitute for the oscillating saw in THA.

## Methods

### Instrumentation design and model study

**Fig. 1:**
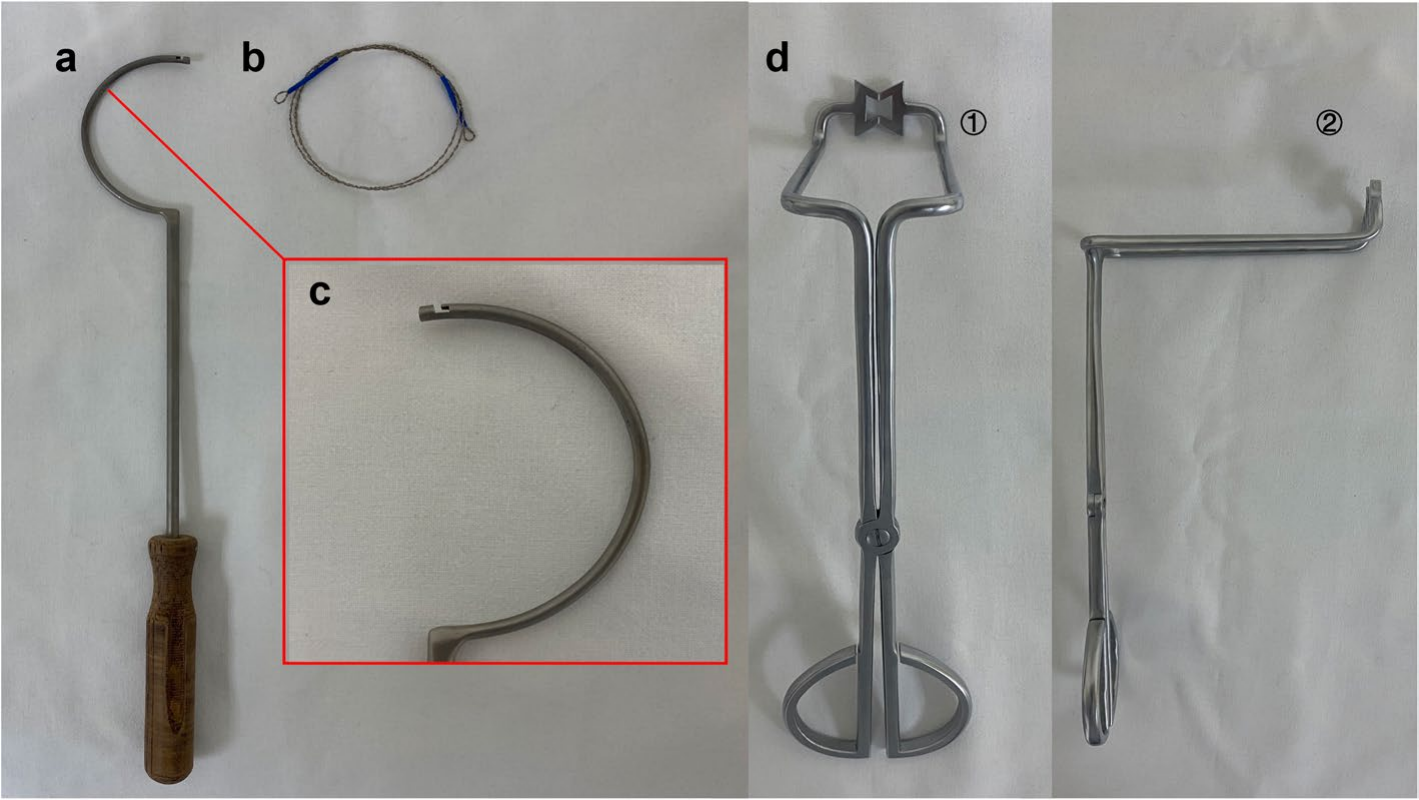
Femoral neck osteotomy instrumentation. **a** The cable passer hook. **b** Fretsaw. **c** Special fillister at the distal end. **d** Anterior view of the jig (①) and lateral view of the jig (②)

**Fig. 2:**
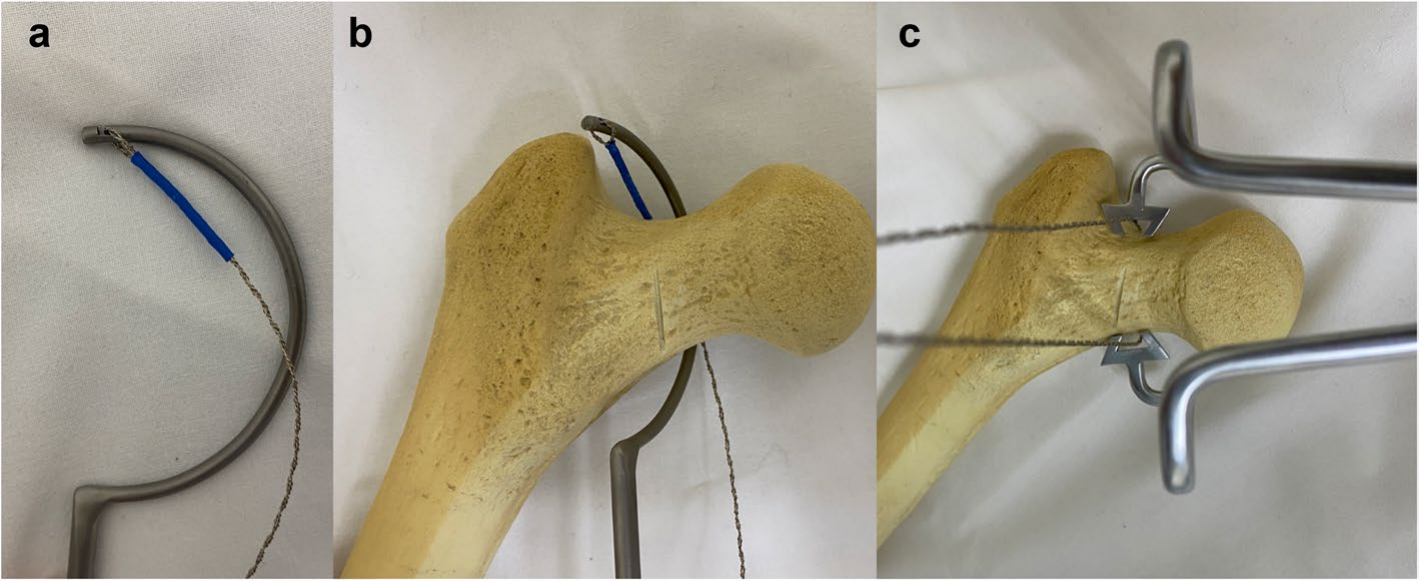
Photograph of the osteotomy instrumentation as a femoral neck osteotomy tool in vitro model. **a** The fretsaw hooking on the terminal of the cable passer hook. **b** Demonstration of the basic principle of the fretsaw placement around the femoral neck. **c** The jig fixing the fretsaw in place for osteotomy

The osteotomy instrumentation (Fig. 1) consists of three separate devices: the fretsaw, cable passer hook, and jig. The fretsaw is also called a wire saw, which is convenient for sterilization and fast replacement of damaged components and is cost-efficient. The cable passer hook was used to carry the fretsaw to round the formal neck. This cable passer hook is a stainless-steel instrument that contains a curved tube and a special fillister at the distal end (Fig. 1c). The fretsaw can be conveniently inserted into this L-shaped fillister and surround the bone. The cuspidal terminal with a groove for the fretsaw and bending handle comprises the jig (Fig. 1d). The perpendicular handle could provide a satisfactory operation field for surgeons without obstruction during visualization. The jig could seize the femoral neck, hold the fretsaw in place for the osteotomy, and prevent cutting damage to the surrounding tissue. To verify the functional demands for the design criteria, a performance experiment was performed using this new osteotomy system on the formal model (Fig. 2). After we confirmed the duration of the procedure, osteotomy height, and quality of cut in the model study, we decided to proceed with the experiment involving the patients.

### Patients

This single-center retrospective study included 40 consecutive patients (40 hips) requiring primary THA in our institution between January 2020 and June 2020. The ethic committee of Shanghai Ninth People’s Hospital approved the study and waived the need for informed consent, since only the human data were collected anonymized. Patients who met the following criteria were included: (1) patients aged between 25 and 80 years; (2) patients with femoral head necrosis, primary osteoarthritis, or rheumatoid arthritis; and (3) surgical procedure via the posterolateral approach using a cementless press-fit cup. The exclusion criteria were as follows: severe hip ankylosis, femoral neck fracture or deformity of the femoral neck, or loss to follow-up. All patients signed a routine operation consent form before undergoing THA surgery and agreed to the potential publication of the collected surgical data. The patients were divided into two groups based on the method of osteotomy. In the new osteotomy instrumentation group, 20 patients underwent femoral neck osteotomy with our designed tool, while the other 20 patients in the oscillating saw group underwent femoral neck osteotomy with an oscillating saw.

### Surgical technique

**Fig. 3:**
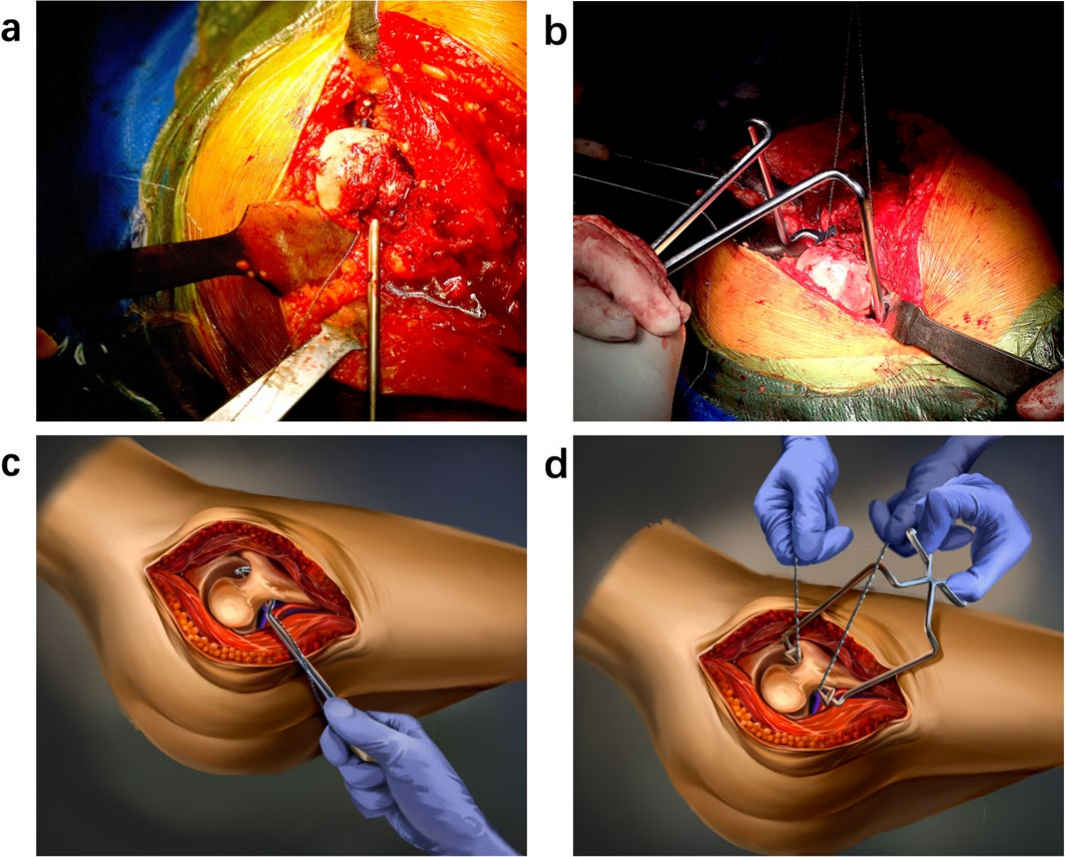
Intraoperative photographs obtained during the surgery. **a, b** Intraoperative photographs of the new osteotomy instrumentation group. **c, d** Diagrams of the osteotomy using the new osteotomy instrumentation

All surgeries were performed by the same surgeon using a posterolateral approach under general anesthesia. The femoral neck osteotomy was implemented according to the preoperative scheme using either a conventional oscillating saw (group I) or the new osteotomy instrumentation (group II) (Fig. 3). The remaining perioperative procedures were identical between the two groups. The rehabilitation programs in the two groups were equal: full weight-bearing at 2 days after surgery.

### Evaluation method and follow-up

Clinical data were obtained from all patients before surgery and at follow-up examinations. Clinical evaluation was performed based on the Harris Hip Score (HHS) [8], and patients’ pain was subjectively described using the visual analogue scale (VAS) [9]. Intraoperative findings, including operation time, osteotomy time, and amount of bleeding, were recorded.

### Statistical analysis

SPSS software (Version 24; SPSS Inc., Chicago, IL, USA) and Prism (Version 8; GraphPad Software Inc., San Diego, CA, USA) were used for statistical analyses. Data were expressed as absolute mean values, ranges, and standard deviations. The distribution of the variables was tested for normality using Student’s t-test, and the Mantel–Haenszel chi-square test was used to compare the categorical variables of clinical results. Difference was considered statistically significant at a *P*-value of < 0.05.

## Results

**Fig. 4:**
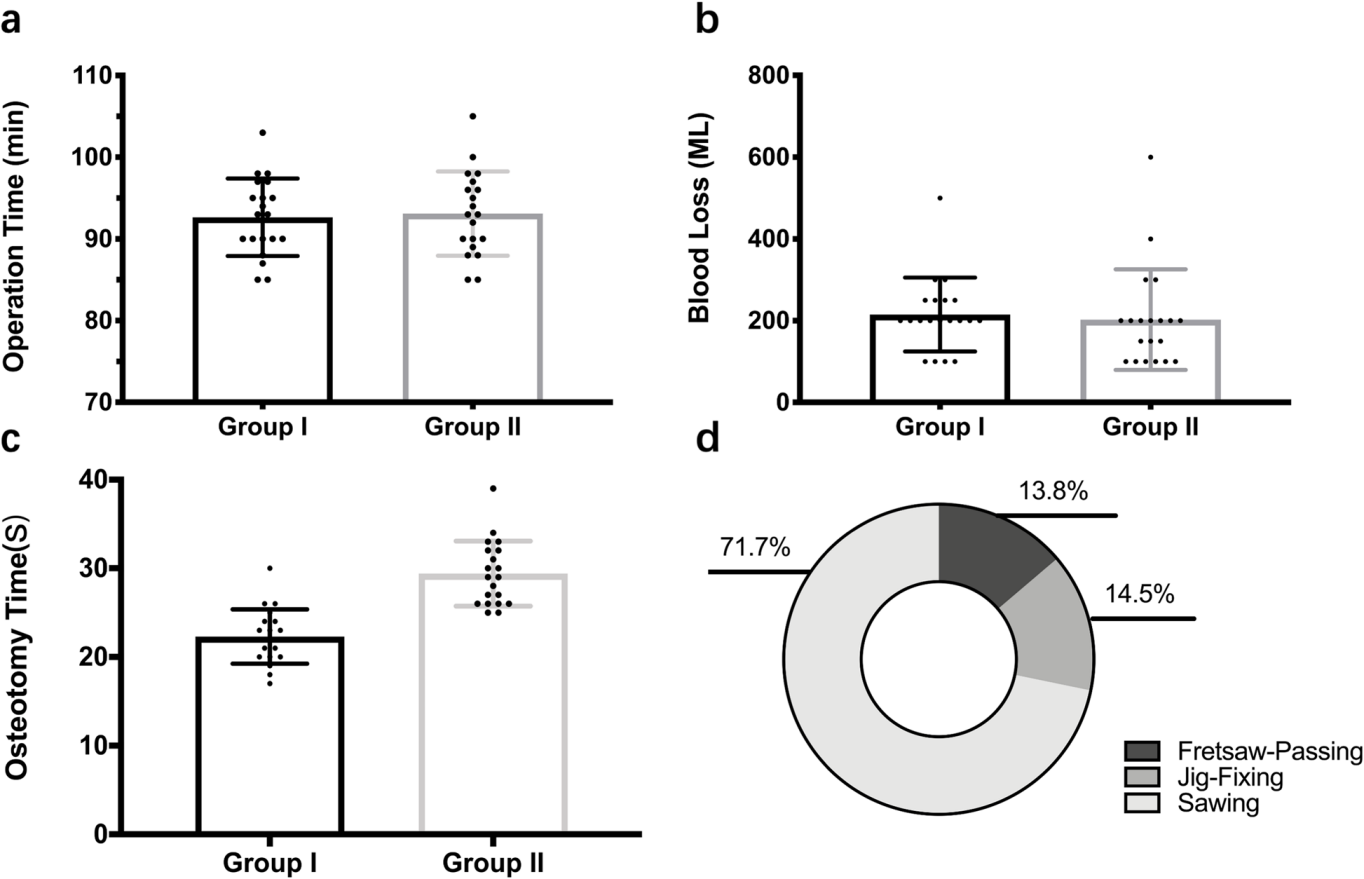
Intraoperative evaluation of the two groups. **a** Total operation time of the two groups. **b** Intraoperative blood loss of the two groups. **c** Osteotomy time of the two groups. **d** Timeline of sectioning motion conducted on the osteotomy in Group II

A total of 40 patients participated in this study, with 20 patients each in the conventional oscillating saw group (group I) and new osteotomy instrumentation group (group II). No patients were lost to follow-up. The clinical characteristics of the patients in the two groups are presented in Table 1. Data on sex, age, body mass index, operative side, and etiology were collected, and the results showed no significant difference between group I and group II. The intraoperative evaluation showed no significant difference in the total operation time (*P* = 0.775) and blood loss (*P* = 0.716) for both groups, while differences existed in the osteotomy time between the two groups (*P* < 0.001); the average osteotomy time in the new osteotomy instrumentation group was longer than that in the oscillating saw group. In addition, the osteotomy time in group II consisted of the fretsaw-passing time (4.05 ± 0.76 s), jig-fixing time (4.25 ± 0.72 s), and sawing time (21.10 ± 3.60 s) (Fig. 4**)**. However, no difference was observed in the sawing time between the two groups (*P* = 0.263). No complications occurred in either group during the osteotomy process in patients undergoing THA.

**Table 1:** Characteristics of patients in the Group I and Group II

	**Group I(*N* = 20)**	**Group II(*N* = 20)**	***P* value**
**Gender**			0.337
Male	10	7	
Female	10	13	
**Mean age(yrs)**	62.4(range,27–78)	56.8(range,26–71)	0.154
**BMI**			0.736
> 25	7	6	
≤ 25	13	14	
**Operation side**			
Right	9	10	0.752
Left	11	10	
**Aetiology of indications**			0.527
Femoral head necrosis	11	9	
Primary osteoarthritis	9	11

A significant improvement in the HHS and VAS score after surgery was observed in the two groups (Table 2**)**. In the oscillating saw group, the mean HHS improved from 44.5 points (standard deviation [SD], 4.7; range 30–52 points) to 82.3 points (SD, 2.5; range 78–90) at the 6-month follow-up examination (*P* < 0.001). In the new osteotomy instrumentation group, it improved from 46.2 points (SD, 4.5; range, 40–55 points) to 83.3 points (SD, 3.5; range, 75–88 points) at the 6-month follow-up examination (*P* < 0.001). The mean preoperative and postoperative VAS for the oscillating saw group was 5.3 points (SD, 0.9; range, 4.0–7.0 points) and 1.4 points (SD, 0.6; range, 0.0–2.0 points), respectively. The mean preoperative and postoperative VAS for the new osteotomy instrumentation group was 5.0 points (SD, 0.8; range, 3.0–6.0 points) and 1.3 points (SD, 0.8; range, 0.0–2.0 points), respectively. There was no significant difference in the VAS score and HHS between the two groups (*P* = 0.651 and *P* = 0.297, respectively). During the follow-up period, no cases of dislocation or infection were observed in any of the groups.

**Table 2:** Comparison of the clinical results between Group I and Group II

	**Group I(M ± SD)**	**Group II(M ± SD)**	**t Value**	***P* Value**
Pre-OP HHS	44.5 ± 4.7	46.2 ± 4.5	1.114	0.246
Post-OP HSS	82.3 ± 2.5	83.3 ± 3.5	0.940	0.297
t Value	32.03	29.05		
P Value	P < 0.001	P < 0.001		
Pre-OP VAS	5.3 ± 0.9	5.0 ± 0.8		0.361
Post-OP VAS	1.4 ± 0.6	1.3 ± 0.8	0.462	0.651
t Value	14.42	14.41	0.960	
P Value	*P* < 0.001	*P* < 0.001		

*M* Mean, *SD* Standard deviation

## Discussion

This study introduced a novel osteotomy instrumentation (fretsaw, jig, cable passer hook) as a substitute tool while the oscillating saw was unavailable during THA. To the best of our knowledge, this is the first substitute device that requires low-tech cleaning and sterilization within an acceptable time frame and yields satisfying osteotomy results, as well as it meets the functional requirements, in THA surgery.

Several studies have shown that long operative times are associated with perioperative complications. Prolonged operation time can increase the risk of blood loss and periprosthetic joint infection, which may be associated with extended hospitalization, financial hardship, and even risk of mortality among patients [10–13]. In this study, the average osteotomy time was a little longer in the new osteotomy instrumentation group than in the oscillating saw group, yet there was no significant difference in the total operation time between the two groups. The osteotomy instrumentation performed efficiently as an oscillating saw and did not prolong the entire operation time. Thus, this fully functional, detachable, and flexible instrumentation could be the ideal choice for osteotomy when the oscillating saw is deactivated. Besides, complications have occasionally been reported during THA, which includes bleeding, soft tissue damage, and fractures [14]. The major sources of bleeding in THA are the ischiofemoral ligament and posterior labrum; bleeding may occur if the surgeon performs an aggressive femoral neck osteotomy using an oscillating saw [15]. The inadvertent penetration of the oscillating saw into the soft tissue leading to neurovascular branch damage may cause postoperative pain and neural and vascular complications, although the reported rate of severe damage is rather low [16]. Unlike the oscillating saw, which requires force toward the bone and the tissues from above, the fretsaw is placed around the bone and directed away from the adjacent soft tissue through the bone. Thus, there is no risk of overshooting or surrounding tissue and trochanter damage during the osteotomy. In addition, the use of this instrumentation resulted in a flat osteotomy surface without the risk of notch generation and fracture of the ipsilateral trochanter. Therefore, it may offer a safe method of femoral neck osteotomy with a reduced risk of trapping soft tissue and periprosthetic femoral fracture.

The major objectives of THA include postoperative improvement of self-reported physical functioning, pain relief, and quality of life [17]. In this study, improvement in the HHS and VAS score was observed in both groups. There was no significant difference in the postoperative HSS and VAS score between the oscillating saw group and the new osteotomy instrumentation group at the 6-month follow-up (P > 0.05). These results demonstrated that the new osteotomy device could be used as a novel osteotomy technique in THA, which had no influence on physical rehabilitation and pain subsided.

However, there are still great challenges for surgeons to deal with complicated hip joint disease caused by congenital disease, rheumatic disease, and other serious diseases [18]. In these cases, the fused hips are difficult to dislocate and reconstruct accurately. The surgeon may encounter a struggle with an inconvenient osteotomy position and a higher risk of neurovascular branch damage during the cutting process, which can prolong the entire operation time. With our newly designed instrumentation, the femoral head does not have to be dislocated prior to osteotomy, since the fretsaw could easily surround the femoral neck through the cable passer hook. In this case, osteotomy can easily be performed in situ with no need to excessively release the soft tissues, which results in less bleeding, enhanced stability, and faster rehabilitation [19].

Over the decades, many research studies have been conducted to explore and advance THA. In the thousands of study topics in this therapeutic method, surgical methods, postoperative outcomes, and materials remain the major focus [20]. However, thus far, this study is the first to focus on the femoral neck osteotomy tool, and introduces and evaluates whether this novel osteotomy instrumentation could efficiently accomplish femoral neck osteotomy during THA surgery. Our clinical data and follow-up outcomes show that the combination of the fretsaw, jig, and cable passer hook can accomplish a safe, minimally invasive, efficient, and precise femoral neck osteotomy.

In many developing and less developed countries, the distribution of medical resources is uneven. Moreover, in several small-scale hospitals, there might be a limitation on the usage of a substitute oscillating saw or matching batteries in the operating room, which could be time consuming in terms of repeating the sterilization of the oscillating saw or acquiring a new oscillating saw from the medical manufacturer. In such situations, the malfunctioning machine and extended operation time could be detrimental for the patient and surgeon. However, our osteotomy instrumentation could be sterilized as a conventional surgical instrument and stored for a long period, thus making it readily accessible as a substitute tool when the oscillating saw is unavailable.

Nevertheless, this study had several limitations. First, the small sample size of the study resulted in a low generalizability of conclusions. Therefore, the study findings still require further verification. Second, the jig could not fix the femoral neck sufficiently, which required a reformative jig with acumination. Consequently, the osteotomy instrumentation needs to be modified for the surgery. Third, the osteotomy tools in both groups were only used in patients with a relatively normal femoral neck. Patients with severe hip ankylosis, femoral neck fracture, or deformity of the femoral neck were excluded from this study. Thus, randomized controlled trials with larger sample sizes, higher quality, and a longer follow-up period are warranted to confirm the results.

## Conclusions

This study demonstrated that the performance of the novel and innovative osteotomy instrumentation was equal to the conventional oscillating saw during THA in terms of operation time and postoperative physical function. Thus, this osteotomy instrumentation serves as an ideal substitute tool for femoral neck osteotomy during THA when the oscillating saw is unavailable or malfunctioning. Furthermore, studies with modified tools, larger samples, and long-term results are required in the future to clarify these findings.

## Data Availability

The datasets generated and/or analysed during the current study are available in the Aliyundrive repository (https://www.aliyundrive.com/s/C1G1xuGbbcw).

## Abbreviations

THA: Total hip arthroplasty
HHS: Harris Hip Score
